# Supplementary material for: Are carbon emissions trading and green financial instruments synergistic? -Comprehensive quantitative research based on content analysis
Source: PLoS One. 2024 Mar 7;19(3):e0298601. doi: 10.1371/journal.pone.0298601 (PMC10919601; doi:10.1371/journal.pone.0298601)
Supplement: S1 Data — (ZIP) [file pone.0298601.s001.zip › Data/地级市Z_F工作报告.zip!/╕ú╜¿/┴·╤╥╩╨/└┤╫╘╣1⁄2╓┌║┼í╛┬φ┐╦╩2╛▌═°í┐_2012.docx]

各位代表：

　　现在，我代表市人民政府向大会作工作报告，请予审议，并请市政协委员和各位列席人员提出意见。

　　一、过去五年的工作

　　市三届人大一次会议以来，我市各级政府在省委、省政府和市委的正确领导下，坚持以科学发展观为统领，认真贯彻胡锦涛总书记考察福建和我市时的重要讲话及重要回信精神，按照中央支持海西建设和省委、省政府加快福建科学发展、跨越发展的战略部署，全面落实市委构建“三大体系”、发展“两大经济”的重大决策，突出集中精力抓工业、集中力量上项目、集中智慧解难题、集中财力办大事，有效应对国际金融危机等不利影响，扎实推进生态型经济枢纽和海西重要增长极建设，顺利完成了市三届人大一次会议确定的主要目标任务，实现了“十二五”发展的良好开局。

　　1、加快转变、提质扩量，综合实力明显提升。初步统计，2011年全市地区生产总值突破1000亿元，达到1285亿元，是2006年的2.7倍，总量从全省的第六位提升到第五位；财政总收入突破200亿元，达到206.4亿元，是2006年的2.7倍。二、三产业占三次产业的比重由2006年的82.7%提高到87.6%。规模以上工业总产值达到1633亿元，是2006年的4.2倍，规模工业经济效益综合指数连续8年居全省首位。培育了6个百亿产业、4个百亿园区、2家百亿企业，新增产值亿元以上工业企业120家、累计315家，新增上市企业6家、累计9家。龙岩经济开发区申报国家级经济技术开发区取得实质性进展。永定客家土楼成功列入世界遗产名录，新增1个国家5A级景区、3个国家4A级景区，全市旅游人数突破1000万人次、达到1163万人次，实现旅游总收入85亿元、是2006年的2.7倍。实现社会消费品零售总额373亿元、是2006年的2.5倍，物流业增加值75亿元、是2006年的2.7倍。金融机构人民币存、贷款余额分别达到910亿元、880亿元，是2006年的2.6倍和3.3倍。成功创建国家可持续发展实验区和可持续发展产业示范基地，荣获全国科技进步先进市称号。单位生产总值能耗降幅和淘汰落后产能总量居全省首位，主要污染物总量减排任务如期完成。

　　2、用好政策、突破项目，发展空间明显拓展。争取原中央苏区参照执行西部地区政策取得突破，我市可参照的83项政策，已落实66项，累计争取中央财政补助资金55.5亿元。“五大战役”和“工业发展年”、“项目建设年”活动成效显著，“三维”项目对接有力推进。五年累计完成全社会固定资产投资2348亿元，是前五年的4.6倍。累计实施投资亿元以上重点项目564项，其中投资10亿元以上项目105项，近三年来我市列入省重点项目的项目数、总投资额、年度投资额均居全省前列。龙工挖掘机、龙工精密铸锻件、新龙马年产5万辆中重卡、精品“七匹狼”卷烟专用生产线、紫金山金铜矿联合开发、紫金20万吨铜冶炼、坑口火电厂二期、漳平电厂改扩建等一批重大生产性项目建成投产。建成龙长、永武高速公路和双永高速公路新罗至永定段，新增高速公路349公里，累计391公里；开工建设双永、蛟城、湖城、古武高速公路和龙厦快速铁路、赣龙铁路扩能工程、龙岩通用直升机综合服务基地等一批重大交通项目。新增电力装机容量55.4万千瓦，累计335.4万千瓦。

　　3、强化统筹、夯实基础，城乡面貌明显变化。新农村建设扎实推进，强农惠农政策得到有效落实，五年来市级投入支农资金11.8亿元。在全国率先启动大规模生猪标准化养殖工程。在全省率先开展农田基本建设整村推进工程，比全省提前三年完成初级水利化县建设，在全省山区市中率先实现村村通水泥路。完成植树造林206.7万亩，森林覆盖率保持全省首位。农村劳动力转移实现“双百”目标，累计转移农村劳动力100.6万人，年劳务收入超过150亿元。城镇化率达46.6%，比2006年提高7.1个百分点。中心城市带动作用增强，规划体系更加健全；市政项目累计完成投资59.8亿元，建成人民会堂、博物馆、第二水源、东风高架桥、莲花山木栈道等一批公用设施，新增城市道路36.6公里、污水管网36.2公里；商务、商贸物流、人居三大板块建设加快推进，商务营运中心基本建成，以闽西交易城、龙门物流园区、龙岩陆地港为主体的中国闽西商贸新城初步形成，莲东、龙腾人居板块初具规模；“绿亮美”工程深入实施，累计完成投资12.3亿元；建成区面积新增8.4平方公里，达到40平方公里；荣获省级文明城市、卫生城市、园林城市称号，创建国家园林城市通过实地验收。县域经济快速发展。小城镇建设稳步推进。

　　4、先行先试、扩大开放，经济活力明显增强。政府机构改革不断深化。行政审批制度改革走在全省前列，行政服务标准化成为国家级试点。“三警合一”警务体制改革顺利实施。中心城市相对集中行政处罚权工作有序推进。成功实施“三资”运作，新组建的“三大集团公司”总资产达168亿元，2011年实现利润2.2亿元。在全省率先试行编制国有资本经营预算。农村工作机制不断创新，集体林权制度改革持续深化。土地承包经营权流转率达39.6%，居全省首位。医药卫生和科教文化体制改革扎实推进。商务综合行政执法试点考评得分居全国第一。外贸出口总值达到17.1亿美元，是2006年的11倍，总量从全省第九位提升到第六位，被评为全国外贸百强城市。成为东部地区唯一的国家加工贸易梯度转移重点承接地。五年累计实际利用外资14.5亿美元，内联引资实际利用市外资金507.6亿元。龙台交流合作进一步深化，海峡两岸机械产业博览会升格为国家级展会，漳平（永福）台湾农民创业园升格为国家级台湾农民创业园，“海峡客家”成为海峡两岸交流合作的重要品牌。

　　5、以人为本、民生优先，人民生活明显改善。组织实施 “五大民生工程”，大力推进“民生龙岩”建设。各级财政五年累计用于民生支出281亿元，年均增长27.2%；其中市级财政支出62.2亿元，民生支出占市级新增财力的70.5%。实施了101个为民办实事项目，累计投入资金48亿元。城镇居民人均可支配收入和农民人均纯收入分别达到20980元和7970元，分别比2006年增加9262元和3479元。成为我省首个省级创建创业型城市试点。社会保障体系基本形成，城乡低保应保尽保，提前三年实现“新农合”制度全覆盖，在全国率先推出“新农合”大病补充医疗保险和“三农”综合保险，在全省率先实现新农保全覆盖、第二个实现城镇居民养老保险全覆盖，提前两年实现国家基本医保目标。五年新建保障性住房151万平方米。“造福工程”搬迁4.7万人。革命基点村和少数民族乡村生产生活条件不断改善。实现每个乡镇至少建成1所公办幼儿园，提前两年实现“双高普九”目标，高中阶段教育走在全省前列，闽西职业技术学院、龙岩技师学院成为国家级职业教育骨干或示范建设单位，龙岩学院稳步发展。校安工程建成面积53.4万平方米。古田会议旧址群等历史文物保护得到加强，公共文化服务设施建设力度加大，《红色摇篮》、《土楼神韵》等文化精品不断涌现。新建和改造乡镇卫生院102所，新组建市妇幼保健院，建成市中医院病房大楼，公共卫生服务体系建设不断加强。体育基础设施建设跨入全省先进行列，我市运动员在北京奥运会、第十一届全运会上所获金牌总数均居全省首位。被确定为全国人口和计划生育综合改革示范市。妇女儿童发展“两纲”主要指标全面完成。全省最大的市福利中心一期、市妇女儿童活动中心一期投入使用。组织实施环保治理“三大工程”，“三江”流域水质达标率高于省定目标，中心城市空气质量优良以上天数达357天、比2006年增加70天。长汀水土流失治理成效显著。防灾减灾工作进一步加强。安全生产“四项指数”连续五年全面下降。食品药品安全监管力度加大。完成“五五”普法。成为全省首个全部县（市、区）通过平安验收的设区市。荣获全国“双拥”模范城称号。

　　6、依法行政、廉洁从政，政府自身建设明显加强。制定实施加强法治政府建设的意见，大力推进依法行政。自觉接受市人大及其常委会的法律监督、工作监督和市政协的民主监督，广泛接受社会监督。认真听取各民主党派、工商联、人民团体、无党派人士和社会各界人士的意见建议，加强与工会、共青团、妇联、科协等人民团体的联系。市三届人大一次会议和市政协三届一次会议以来交由政府系统承办的1126件市人大代表建议、批评、意见和1930件市政协提案全部办结，满意率持续提升。政府绩效管理全面推行，电子政务、政务公开和政府信息公开深入推进，政府绩效评估和投资环境监测评估在全省名列前茅。坚持“两个务必”，强化廉政建设，行政监察、审计监督和纠风工作进一步加强。

　　回顾过去五年，我们深切体会到，做好政府工作，必须坚持以科学发展观为统领，好字当头、好中求快、又好又快，在转变中谋发展，在发展中促转变；必须坚持以解放思想、先行先试为动力，用开放的视野拓宽发展思路，用改革的冲劲突破体制机制障碍，用创新的举措解决发展难题；必须坚持以项目带动为抓手，突出抓大项目、上好项目，以项目扩总量、调结构、增后劲、惠民生；必须坚持以保障和改善民生为目的，真正把民生摆在优先位置，体察民情、化解民忧、维护民利，做到改革发展成果由全市人民共享；必须坚持以提高行政效能为保障，不断加强政府自身建设，提升各级政府和政府工作人员的学习力、凝聚力、执行力、公信力，为做好政府各项工作提供坚强保证。

　　各位代表，过去五年，是龙岩综合实力提升最快、城乡面貌变化最大、人民群众受益最多的历史发展时期之一。这是省委、省政府和市委正确领导的结果，是全市人民齐心协力、奋力拼搏的结果，是社会各界广泛参与、大力支持的结果。在此，我代表市人民政府，向为龙岩发展付出辛勤劳动的全市广大干部群众；向给予政府工作有力支持与有效监督的人大代表和政协委员、各民主党派、工商联、人民团体、无党派人士、离退休老同志和社会各界人士；向为龙岩改革发展稳定作出积极贡献的中央和省直驻岩机构、驻岩部队、武警官兵、公安政法干警；向所有关心支持龙岩发展的港澳台同胞、海外侨胞、国际友人，表示衷心的感谢和崇高的敬意！

　　在肯定成绩的同时，我们也清醒地认识到存在的问题与不足。主要是：经济总量偏小；转方式、调结构进展不平衡，自主创新能力不强；资源环境约束加剧，节能减排任务艰巨；经济外向度较低；城乡区域发展不够协调，农民持续增收难度较大；基本公共服务水平与人民群众的期待还有差距，社会管理存在薄弱环节；一些政府工作人员的思想观念、知识结构、执行力还不适应推进科学发展、跨越发展、加快崛起的要求，消极腐败现象在一些地方、一些领域仍然存在。我们将以高度负责的精神，采取更加有力的措施，切实解决好这些问题。

　　二、新一届政府工作的目标任务和2012年工作安排

　　今后五年，是龙岩实现全面建成小康社会目标的关键时期。省第九次党代会，作出了坚持科学发展跨越发展、为建设更加优美更加和谐更加幸福的福建而奋斗的战略部署，对闽西老区提出了加快崛起的新要求。市第四次党代会，确立了把龙岩建

　　成生态型经济枢纽和可持续发展产业集聚区，走在全国著名革命老区、原中央苏区发展前列的奋斗目标。新一届政府将全面贯彻省第九次党代会和市第四次党代会精神，紧紧抓住中央支持海西建设和原中央苏区发展的重大历史机遇，充分发挥政策、生态、区位、人文、资源等优势，努力实现好中求快、又好又快的发展。

　　今后五年政府工作的指导思想是：高举中国特色社会主义伟大旗帜，以邓小平理论和“三个代表”重要思想为指导，深入贯彻落实科学发展观，按照省第九次党代会、市第四次党代会的部署，以科学发展为主题，以加快转变、跨越发展为主线，以经济结构战略性调整为主攻方向，以保障和改善民生为立足点，以打造“红土政风”品牌为保障，加快构建产业、基础设施、城市“三大体系”，发展新农村、民生“两大经济”，建设产业龙岩、创新龙岩、生态龙岩、文化龙岩、民生龙岩，提升农业现代化、新型工业化和城镇化水平，提升人民群众幸福指数，决战全面建设小康社会目标，把龙岩建设成为生态型经济枢纽和可持续发展产业集聚区，在福建发展和海西建设中加快崛起，使闽西老区山更青、水更绿、人民更安康。

　　主要目标：力争实现“三个高于”，即主要经济指标增幅、城乡居民收入增幅、单位生产总值能耗降幅均高于全省平均水平。到2015年实现“五个突破、五个翻番”，力争到2016年，全市地区生产总值达到2200亿元以上，规模以上工业总产值达到3500亿元以上，全社会固定资产投资五年累计达到6000亿元以上，社会消费品零售总额达到700亿元以上，财政总收入达到400亿元以上。

　　（一）着力城乡统筹，在推进城乡一体化发展中加快崛起。按照“规划一体化、基础设施一体化、基本公共服务一体化”的要求，统筹推进新农村建设和中心城市、县域城市、小城镇三级城市体系建设，努力实现城乡协调发展、共同繁荣。

　　加快建设社会主义新农村。全面落实强农惠农富农政策，健全以工促农、以城带乡的长效机制，力争2016年全市农民人均纯收入超过全省平均水平。毫不放松抓好粮食生产。积极发展精细农业、设施农业和高效生态农业，做大农业特色产业和特色产品。大力培育农业产业化龙头企业和农民专业合作社，加快建设一批农产品加工专业园区。加强农产品质量安全监管体系和重大动植物疫病防控体系建设，提高农产品质量。加大农业科技创新和推广力度，加强农村“六大员”队伍建设，健全农村社会化服务体系。加快发展农村物流业、旅游业，促进一产和三产融合发展。用现代职业教育方式培育新型农民，多渠道转移农民就业。抓好新一轮扶贫开发，实施老区村跨越发展工程。重视发展壮大村集体经济。加大农村基础设施投入，实施新一轮水利建设，落实十年200亿元投资计划，确保2014年全面完成农村饮水安全工程建设，构建强有力的综合水利保障体系；推进农村公路和电网改造升级，加强养护管理；健全防灾减灾体系。提升新农村建设规划，加强农村环境综合整治，加快建设中心村和新型农村社区，打造宜居新村。

　　加快打造海峡西岸经济区西部中心城市。突出红色、客家、生态文化特色，完善城市规划，增强规划的前瞻性、系统性、特色性和权威性。呼应海西城市群发展，主动融入厦漳泉大都市区，继续推动厦泉漳龙城市联盟建设。积极推进行政区划调整，努力实现“一市多区”。按照“一主三副”的城市布局，高起点规划建设管理中心城市主城区和高坎、龙雁、古蛟三座新城。实施“畅通工程”，加快中心城区“一条主轴、一个外环”的交通主干道路和连接高坎、龙雁新城的城际干线建设，完善城市路网，大力发展公共交通，构建方便、快捷的城市综合交通体系。大力推进中心城市“五大板块”建设，加快“六个一”工程和一批城市综合体建设，完善城市服务功能，构建现代都市核心区。统筹推进新区开发与旧城改造，深入实施“绿亮美”工程，强化城市管理，大力提升城市品位。严格城市综合执法，有效制止违法违章建设。

　　加快发展县域经济。突出县域经济特色，强化县域发展功能，壮大县域产业实力，增强县域城市的承载力、集聚力和带动力。力争到2015年，新罗综合经济实力在全省各区排位进一步前移；上杭、永定、漳平进入全省经济较发达县（市）行列；连城、长汀在全省中等发展水平县（市）的排位进一步前移；武平进入全省中等发展水平县（市）行列。加快推进15个省、市综合改革建设试点镇发展。加强中心镇建设，力争打造30个工业强镇、20个商贸重镇和10个旅游名镇。积极开展撤乡并镇试点。加大政策引导和推动力度，促进边远山区农民向城镇集聚。

　　（二）着力产业升级，在推进可持续发展产业集聚区建设中加快崛起。围绕建设“产业龙岩”，把调整优化产业结构作为加快转变经济发展方式的主攻方向，依托国家可持续发展实验区和可持续发展产业示范基地，积极对接全省重点产业和临港工业布局，加快培育“1+2”主导产业，全力打造“155”产业基地，力争到2016年培育1个1000亿级产业、2个500亿级产业、9个100亿级产业，3个500亿级园区、5个100亿级园区和5家100亿级企业。

　　集聚发展主导产业。以龙工、龙净、新龙马等领军企业为龙头，加快推进龙工配件园、龙净工业园、汽车工业园建设，打造世界级工程机械制造基地、全国环保产业基地和海西汽车产业基地，力争2016年机械产业年产值超过1100亿元，其中汽车产业年产值超过500亿元。加快建设上杭铜产业循环经济园，打造全国金铜产业基地，力争2016年有色金属产业年产值超过600亿元，其中铜产业年产值突破500亿元。围绕建设中国闽西商贸新城，继续抓好龙岩陆地港、龙门物流园区等项目建设，全力推进中国（龙岩）农产品物流交易城建设，大力发展会展经济，力争2016年中心城市现代商业集群交易总额超过600亿元，全市物流业增加值达到100亿元以上。

　　大力发展战略性新兴产业和现代服务业。坚持“五统一”原则，发挥厦钨金龙公司引领作用，加快建设龙岩（长汀）稀土工业园，打造全国稀土产业基地，力争2016年稀土产业年产值突破200亿元。加强政策引导，大力发展新材料、新能源、生物与新医药等战略性新兴产业，做大做强新罗硬质合金产业园、连城新材料产业园等专业园区，力争2016年新材料产业年产值突破300亿元。规划建设龙岩研发园，推广应用物联网、云计算等先进技术，建设“智慧城市”。围绕“海峡旅游”主题，重点打造上杭古田、永定客家土楼、连城冠豸山、长汀国家历史文化名城四大旅游景区，完善旅游配套设施和服务体系，打造中国最佳旅游目的地。优化金融发展环境，吸引各类金融机构来岩设点展业，力争设立龙岩银行，在组建红土地创业投资基金的基础上进一步构建创业投资基金体系，有效防范金融风险，促进金融业稳健发展，力争2016年金融机构人民币存、贷款余额均突破2000亿元。加快发展信息服务、商务服务、服务外包等现代服务业，大力发展家政、养老、社区服务等家庭服务业。

　　提升发展传统产业。优化卷烟结构，完善产业链条，打造全国精品卷烟生产基地。提高新型干法水泥比重，稳步发展陶瓷工业，引进开发新型建材，打造全国建材产业基地。加强纺织企业协作配套，培育做大品牌，打造海西纺织产业基地。实施“西气东输”经龙岩引入福建项目，推进天然气发电等后续项目开发建设，打造海西能源基地。加快建设漳平特钢铸造产业园和武平不锈钢产业园。

　　（三）着力先行先试，在推进改革开放中加快崛起。围绕建设“创新龙岩”，把解放思想、创新创造贯穿于推动发展的全过程，努力实现改革开放的新突破。

　　深化体制改革。进一步下放市对县（市、区）经济社会管理权限，推进扩权强镇。继续抓好农村综合改革，开展城乡统筹综合配套改革试验，深化集体林权制度配套改革，力争2016年农村土地承包经营权流转率达到50%。完善财政预算体系，健全地方政府债务管理机制。继续推进国有资产管理体制改革。深化“三资”运作，力争2016年“三大集团公司”总资产和利润分别突破500亿元和15亿元。分类推进事业单位改革。稳步推进科技、教育、文化、卫生等领域改革。创造良好环境，壮大民营经济。创新投融资模式，争取新增上市企业10家。

　　增强创新能力。健全鼓励自主创新的支持服务体系，推动发展由“要素驱动”向“创新驱动”转变，由“龙岩制造”向“龙岩创造”转变，加快建设“创新龙岩”。确保财政对科技投入的增幅高于经常性财政收入的增幅。突出人才第一资源，进一步完善人才工作机制、创新人才使用模式、优化人才成长环境，促进创新人才的培养和引进。确保创建国家级经济技术开发区。继续争创国家高新技术产业开发区，培育具有鲜明特色的创新型产业集群，建设海西现代化科技新城。力争培育高新技术企业100家以上。进一步加强科技成果转化平台建设和知识产权保护。

　　提升开放水平。把实施全方位、高水平的对外开放战略摆上更加突出的位置，在经济全球化、区域经济一体化中拓展发展空间。加强“三维”招商，做到力度更大、办法更活、服务更优、成效更好。进一步发挥国家加工贸易梯度转移重点承接地效应，努力培育10个超亿美元、100个超千万美元的出口企业，力争2016年外贸出口总值突破30亿美元。深化龙港、龙澳合作，密切闽西南五市、闽粤赣十三市和海西中部集中发展区、闽粤赣互动发展区的区域协作，加强外事侨务和海外联谊工作。进一步拓宽对外开放通道，加快推进双永、厦蓉高速漳州天宝至龙岩蛟洋扩容、漳永等高速公路和龙厦、南三龙、浦建龙梅、龙岩经永定至河源等铁路的规划建设， 确保实现2012年县县通高速公路，力争实现2015年县县通快速铁路，到2016年新建高速公路385公里、快速铁路170公里。实施国省道提级改造工程，完善次网骨架。加快推进冠豸山机场扩能改造和龙岩通用直升机综合服务基地建设。

　　密切龙台合作。以海峡两岸机械产业博览会为平台，加强与台湾工商团体联系，深化与台湾机械、光伏、农产品加工等产业对接。以漳平（永福）国家级台湾农民创业园为重点，加快扩园步伐，抓好县域龙台农业合作园区和台商投资产业园建设，争取设立国家级台商投资区。以“海峡客家”品牌为纽带，继续办好福建土楼文化节、海峡客家旅游欢乐节和定光佛文化节，增进对台民间往来，深化科技、教育、文化、卫生等领域的交流合作。

　　（四）着力生态建设，在推进资源节约型和环境友好型社会建设中加快崛起。围绕建设“生态龙岩”，坚持生态优先、环保为民，走经济效益、社会效益、生态效益有机统一的发展之路。

　　加强节能减排。严格执行节能减排目标责任制，严把产业政策关、环境准入关和资源节约关，确保到2015年单位生产总值能耗下降19%，二氧化硫、化学需氧量、氨氮、氮氧化物完成省下达减排任务。突出抓好水泥、造纸、冶金、砖瓦等行业的节能减排。推进中心城市“优二进三”，全面完成10家企业关闭搬迁。大力发展循环经济，推行清洁生产，力争创建1个以上国家级循环经济示范园区。

　　确保青山常在。以创建国家园林城市为新的起点，扎实推进“生态市”建设，力争2015年全部县（市、区）达到生态县标准。加强“森林龙岩”建设，深入实施“四绿”工程，建成以汀江、九龙江、闽江生态公益林为重点的生态安全体系，完成植树造林120万亩以上，保持森林覆盖率居全省首位。总结推广长汀经验，力争对235万亩水土流失区域进行全面治理。严格耕地保护，节约集约利用土地。健全生态补偿机制，推进矿区生态恢复，筑牢生态屏障。

　　实现绿水长流。以创建国家环保模范城市为抓手，全面落实环境保护“一岗双责”制度，突出抓好以流域水环境综合整治为重点的新一轮环境污染治理。加大养殖业污染治理和城镇生产生活污水处理力度，力争用3年时间完成中心城市内河水系整治。加大江河源头和水源地保护力度，确保饮用水源安全。加强工业园区和重点乡镇垃圾、污水处理设施建设，力争2016年全市垃圾、污水处理率分别达到95%和85%。完善污染防控体系，推进城镇大气、噪音、油烟等环境污染和农村面源污染综合整治，增强环境安全突发事件处置能力。

　　（五）着力发挥优势，在推进文化大发展大繁荣中加快崛起。围绕建设“文化龙岩”，坚持先进文化前进方向，增强文化自觉和文化自信，大力推进文化改革发展，提升文化软实力。

　　大力发展文化事业和文化产业。深入实施文化惠民工程，推动公共文化资源向基层延伸、向农村倾斜，加快形成覆盖城乡的公共文化服务体系。鼓励开展形式多样的群众性文化活动。继续实施农村和城市广场公益性电影放映工程。积极发展优秀企业文化。加强文化基础设施建设，扎实推进龙岩动漫科技产业园、科技馆、文化馆、大剧院等文化项目的规划建设，提升乡村和社区文化设施建设水平。加大对文化创意、动漫游戏、工艺美术等文化产业发展的政策扶持力度，加强文化人才队伍建设，培育文化领军企业，力争2016年文化产业增加值占地区生产总值的比重达到8%。推进文化产业与科技、旅游、金融等产业融合发展，加快发展新兴文化业态。大力发展健康向上的网络文化。

　　打造特色文化品牌。充分发掘红色文化、客家文化、闽南文化资源，实施文化精品工程建设。继续抓好古田会议旧址群等重点革命旧址维修保护工程，建设一批红色文化基地，提升闽西红色文化影响力。守护好、传承好、运用好客家文化，争创国家级客家文化生态保护试验区。重视世界文化遗产、非物质文化遗产和地方特色文化的保护传承，加强历史文化名城、名镇、名村、名街的保护开发。

　　争创全国文明城市。深入开展群众性精神文明创建活动，推进“红土地千里文明长廊”建设，争创一批全国、全省文明县城。加强全社会思想道德教育，把社会主义核心价值体系融入精神文明建设，提高公民的整体素质。大力推进社会诚信体系建设。

　　（六）着力民生质量，在推进以改善民生为重点的社会建设中加快崛起。围绕建设“民生龙岩”，顺应人民群众过上更加幸福生活的新期待，深入实施“五大民生工程”，不断提高老区人民的幸福指数。

　　加快完善社会保障体系。实行更加积极的就业政策，实施全民职业技能提升计划，加大力度扶持高校毕业生自主创业，争创“国家级创业型城市”。稳步提高城乡居民特别是低收入群众收入水平。进一步扩大各类社会保险覆盖范围，提高统筹层次和保障水平，力争社会保险全员参保率达到95%以上，覆盖城乡的社会保障体系建设走在全国前列。加强市、县两级福利机构和农村敬老院、社区居家养老服务站建设。加大力度解决城镇农民工在就业、居住、医疗、子女入学等方面遇到的困难，有序引导符合条件的农民工进城落户。建设市、县两级统一便民的人力资源市场和社会保障服务中心。抓好保障性住房建设和管理，新建保障性住房4.8万套、361万平方米。加强对老年人、妇女儿童、残疾人合法权益的保护。加大对革命基点村和少数民族乡村的扶持力度，改善边远山区群众生产生活条件。抓好“菜篮子”工程建设，保持物价稳定。

　　积极发展社会事业。坚持教育优先发展，确保各县（市、区）到2015年全面实现“教育强县”建设目标。力争到2016年，学前三年毛入园率达93%；九年义务教育巩固率达98.5%，中小学校安工程建设全面完成，中心城市、县域城市和中心镇教育稳步扩容，学校布局更加合理，教育资源配置更加科学；高中阶段毛入学率达92%；基本建成与龙岩产业发展要求相适应的现代职业教育体系；高校办学水平明显提升。广泛开展全民健身运动，深入实施“奥运争光”计划，加快推进国家级高水平体育后备人才基地建设，规划建设龙岩体育运动学校，筹建海峡两岸体育交流基地，建设体育强市。加强公共卫生服务体系、重大疾病预防控制体系和卫生监督体系建设，力争优质医疗卫生资源配置走在全省前列，确保2014年创建国家卫生城市。提高全国人口和计划生育综合改革示范市建设水平。

　　加强和创新社会管理。健全信访网络体系和矛盾纠纷排查调处机制。加强流动人口服务管理，加大对特殊人群的帮扶力度，完善信息网络管理体系。加强城乡社区建设，构建和谐劳动关系，夯实社会管理的基层基础。深化“平安龙岩”建设，着力提高对违法犯罪活动的打击、防范、控制能力和突发事件应急处置能力。健全和落实各项安全生产责任制度，有效防范和坚决遏制重特大安全事故的发生。强化食品药品安全监管。推进“六五”普法。深化“双拥”共建，加强人防基地建设，促进军民融合式发展。

　　各位代表，2012年是新一届政府工作的开局之年，也是实施“十二五”规划的关键一年。面对新的形势和任务，市政府将按照“稳中求进”的总基调和“三个高于”的工作要求，全力做好稳增长、调结构、控物价、惠民生、抓改革、促和谐的各项工作。全市经济社会发展的主要预期目标是：地区生产总值增长12%，财政总收入和地方级财政收入均增长16%；全社会固定资产投资增长23%；社会消费品零售总额增长19%；外贸出口总值增长15%；实际利用外资（历史口径）增长10%；城镇居民人均可支配收入增长12%，农民人均纯收入增长12%；居民消费价格总水平涨幅控制在4%左右；城镇登记失业率控制在4.2%以内；人口自然增长率控制在8‰以内；单位生产总值能耗下降3.5%，二氧化硫、化学需氧量、氨氮、氮氧化物完成省下达年度减排任务。我们将确保目标实现，争取更好结果。重点抓好五方面工作：

　　1、抓战役，促投资。确保“五大战役”完成投资800亿元，开工建设400个项目，竣工投产300个项目，策划生成200个生产性项目。打好重点项目建设战役。初步安排重点项目136个，年计划投资264.6亿元。确保龙厦铁路和双永、湖城、蛟城及古武线武平十方至城关段高速公路建成通车，加快赣龙铁路扩能工程、龙岩通用直升机综合服务基地建设，开工建设漳永高速公路漳平段，力争开工建设厦蓉高速漳州金山至龙岩红坊扩容项目。争取新龙马年产15万辆微卡、畅丰车桥异地技改扩建、上杭瓮福紫金磷化工、新罗龙化异地技改扩建、武平坤孚镁合金深加工和漳平特钢铸造产业园一期等项目建成投产，加快新龙马年产30万台发动机、马坑矿业采选扩建工程等项目建设进度，推动新龙马年产15万辆微面、西气东输经龙岩引入福建项目尽快开工建设。打好新增长区域发展战役。八个省级工业园区年计划投资280亿元。争取创建国家级经济技术开发区，继续推进创建国家高新技术产业开发区工作。努力推动龙岩（长汀）稀土工业园、龙雁工业园升格为省级工业园区。加快发展各类特色专业园区。打好城市建设战役。年计划投资162.9亿元。启动实施“点、线、面”攻坚计划。确保龙岩美食城、中央苏区公园一期、石锣鼓湿地公园建成投入使用；加快推进中心城市大外环、中心城区至高新园区城际快速通道和东山等人居板块建设；开工建设龙岩大道三期及高架桥、龙岩展览城；规划建设莲花湖、龙雁快速通道和一批城市综合体。加强城市路网、人行天桥、停车场建设，加大城市交通综合整治力度。加快推进“城中村”、“棚户区”改造，完成850户改造任务。加强背街小巷综合整治。增加普通商品住房供应，促进房地产市场健康发展。打好小城镇改革发展战役。年计划投资65亿元。完成15个省、市综合改革建设试点镇总体规划和重要专项规划编制。全面推进基础设施、公用事业和环境景观项目建设。合理布局城镇产业项目，壮大经济实力。落实试点政策，推进机制创新，增强发展活力。打好民生工程战役。突出抓好重大社会事业项目建设，建成市第二医院分院和市第三医院、龙岩人民医院病房综合大楼，加快推进市第一医院门诊综合楼、妇幼保健院、市妇女儿童活动中心二期等项目建设，规划建设市第一医院分院和龙岩一中分校、北城中心小学。开工建设保障性住房1.2万套。继续实施一批为民办实事项目。

　　2、抓产业，促集聚。抓龙头。落实扶持工业企业发展的政策措施，促进中小企业健康发展。启动实施“龙腾企业”计划，力争新上规模以上工业企业100家，新增产值亿元企业40家、10亿元企业3家。力争龙工（本土）产值突破100亿元。加快培育和发展总部经济。铸链条。重点延伸完善以龙工集团为龙头的工程机械产业链、以龙净公司为龙头的环保设备产业链、以新龙马公司为龙头的汽车产业链、以龙烟公司为龙头的烟草产业链、以紫金矿业集团为龙头的铜产业链、以厦钨金龙公司为龙头的稀土产业链、以金鑫钨业公司为龙头的硬质合金产业链、以鑫晶刚玉公司为龙头的光伏产业链、以赛特新材公司为龙头的新材料产业链、以森宝集团为龙头的农产品加工产业链。力争机械产业产值突破400亿元，有色金属产业产值突破300亿元，建材产业产值突破200亿元，汽车产业和纺织产业产值均突破100亿元；龙岩经济开发区和上杭工业园区产值均突破200亿元。优结构。集中力量实施一批科技重大专项、区域创新和技术改造项目。建成国家空气污染治理设备产品质量监督检验中心。加快龙岩陆地港、龙门物流园区等项目建设，开工建设中国（龙岩）农产品物流交易城。推进长汀国家历史文化名城、永定南溪土楼沟、紫金山休闲体育公园等重大旅游项目建设，做好冠豸山申报世界地质公园工作，力争旅游总收入突破100亿元。加快中心城市金融商务中心建设，积极筹建龙岩银行，规范发展小额贷款公司和融资性担保机构，防范和化解民间借贷风险，确保金融机构人民币存、贷款余额均突破1000亿元。

　　3、抓“三农”，促增收。发展现代农业。确保粮食播种面积268万亩、总产100万吨以上。扩大高效经济作物种植面积。调优养殖结构，创建省级生猪标准化养殖示范场10家，抓好特色养殖。大力培育农业产业化龙头企业，加快发展生猪、肉鸡和薯、茶、竹制品加工，力争实现农产品加工产业产值260亿元以上。强化农产品全程质量安全管理。启动实施新型职业农民培育工程，转移农村劳动力4.7万人。重抓水利建设。力争完成水利投资20亿元，实施47座病险水库除险加固工程，开工和续建16座中小型水库，解决40万以上农村人口饮水安全问题。完善水利设施管护机制。加强扶贫开发。制订新一轮扶贫开发实施意见。完成“造福工程”搬迁4468户、1.8万人。推进第三轮46个省重点扶贫村和53个市扶贫开发重点村整体脱贫。建设宜居新村。突出抓好新农村“427”示范工程建设。实现村庄规划全覆盖。加强农村住房建设管理，加大村庄整治力度，深入实施农村家园清洁行动。开展“一村一社区”建设试点。

　　4、抓改革，促开放。推进改革攻坚。全面完成新一轮政府机构改革。继续推进中心城市相对集中行政处罚权工作。加强乡镇行政服务中心标准化建设。促进农村土地承包经营权规范有序流转。加强预算管理，严格控制“三公”等一般性财政支出，降低行政成本。支持企业发行债券工作，推动龙洲运输、龙马环卫等企业加快上市。认真清理、坚决取消限制民营经济发展的政策规定，大力实施“回归工程”，壮大民营经济。深化医药卫生体制改革。提升开放水平。全面推进与央企、民企、外企“三维”项目对接，用好“6·18”、“9·8”等重大经贸合作平台，拓展新的招商领域，加强个性化、专业化、常态化招商。继续实施鼓励外贸发展政策，力争培育2家超亿美元、50家超千万美元出口企业，外贸出口总量突破20亿美元。承办好第三届“11·8”海峡两岸机械产业博览会。扎实推进创建国家级台商投资区工作。扩大政策效应。认真研究国家和省重大政策走向，善于运用政策，全面落实政策，持续完善政策，努力实现政策效应最大化。

　　5、抓统筹，促和谐。加强生态市建设。确保成功创建国家园林城市。完成植树造林38万亩。抓好以长汀为重点的新一轮水土流失治理，力争治理水土流失区域48万亩。扎实推进汀江、九龙江、闽江流域和中心城市内河水系整治。确保何家陂水库建成蓄水。加快中心城市“优二进三”步伐，力争卓鹰、卓龙、龙化、永峰纸业完成关闭搬迁。加快煤矿企业兼并重组，继续淘汰落后产能。确保市垃圾焚烧发电厂建成投入使用，省级以上工业园区、乡镇工业集中区均建成污水集中处理设施。完成农村环境连片整治试点工作。提升民生保障水平。新增城镇就业2.2万人。健全职工工资正常增长机制和支付保障机制，提高企业职工特别是一线职工工资收入。启动实施城乡居民养老保险待遇倍增计划，全面落实被征地农民养老保障金政策，进一步提高城乡低保标准和优抚待遇。扩大生育保险覆盖范围，完善失业保险制度，实现工伤保险全覆盖。开工建设市老年活动中心。采取综合措施，促进物价稳定。加快发展社会事业。新建、扩建中小学校35所，其中中心城市10所小学。高度重视抓好校安工程，加强学生接送车辆的安全管理。大力支持龙岩学院做好教育部本科教学水平评估工作和闽西职业技术学院建设“国家骨干高职院校”工作，启动建设龙岩技师学院新校区。积极备战奥运会、全运会和城运会。扎实推进创建国家卫生城市工作，抓好县级公立医院综合改革试点，完善基层医疗卫生服务网络。继续做好人口与计划生育工作。推进文化繁荣发展。组建市文化发展集团。承办好福建省第五届艺术节暨第25届全省戏剧会演和福建省苏维埃政府成立80周年纪念活动。争取龙岩动漫科技产业园5月1日开园。加快推进长汀世界客家首府博物馆、永定海峡客家文化城、上杭客家缘文化中心、武平客家文化城、连城海峡客家论坛中心等项目建设。确保社会安定稳定。建立“大调解、大维稳、大服务”工作体系，完善突发事件处置机制，提高信访事件处置能力。深化“平安龙岩”建设，健全社会治安防控体系。加强生产安全和食品、药品安全监管。

　　三、打造“红土政风”品牌，进一步加强政府自身建设

　　各位代表，在更高起点上推进闽西老区科学发展、跨越发展、加快崛起，对政府自身建设提出了新的更高要求。我们将大力弘扬古田会议精神，把发扬老区精神与福建精神结合起来，打造“红土政风”品牌，扎实推进责任政府、服务政府、

　　效能政府、法治政府和廉洁政府建设，为推进龙岩加快崛起提供坚强保障。

　　1、心系群众，服务人民。牢固树立以人为本、执政为民的理念，始终把人民群众放在心中最高位置，把实现好、维护好、发展好最广大人民的根本利益，作为政府一切工作的出发点和落脚点。突出决策为民，更加注重问政于民、问需于民、问计于民，做到政策制订着眼民生，工作部署立足民生。突出发展惠民，更加注重一手抓发展、一手抓民生，做到发展与民生同谋划、同部署、同推进。突出保障利民，更加注重办好一批民生实事，推进一批惠民工程，打造一批“民生龙岩”品牌，描绘更加精彩的民生画卷。

　　2、全力跨越，加快崛起。牢牢把握科学发展主题，紧紧抓住加快转变、跨越发展主线，推动闽西老区加快崛起，是我们的历史使命。我们将大力传承苏区干部“干革命走前头、搞生产争上游”的光荣传统，弘扬“爱国爱乡、海纳百川、乐善好施、敢拼会赢”的福建精神，以“追”和“赶”的韧劲，以“拼”和“闯”的决心，坚持解放思想，敢于先行先试，在改革上放胆、政策上放活、市场上放开、管理上放权，全面激发发展活力，实现跨越，推进崛起。

　　3、真抓实干，务求实效。重抓执行，对议定的事一抓到底，对承诺的事有诺必践，确保中央、省和市委的决策部署落实到位。重抓效能，全面推行政府绩效管理，加强政府督查和行政问责，建立电子监察平台，加大治庸治懒治散力度，切实改进会风、文风。重抓服务，强化“环境是对外开放第一竞争力”的理念，学习借鉴厦航先进服务经验，全面推行行政服务标准化，优化发展环境，提升服务质量。

　　4、依法行政，廉洁从政。更加自觉接受人大及其常委会的法律监督、工作监督和政协的民主监督，强化政府层级监督和监察、审计等专门监督，高度重视社会公众监督和舆论监督。更加重视法治政府建设，提升依法、科学、民主决策水平，深化行政执法体制改革，完善规范性文件监督管理制度，推进政务公开和政府信息公开，进一步提高政府工作人员运用法治思维和法律手段解决问题的能力。更加严格落实党风廉政建设责任制，牢记“两个务必”，特别要在国有资产管理、国有土地出让、探（采）矿权转让、建设工程招投标、政府采购等方面严肃党纪政纪法纪，完善各项机制，坚决纠正不正之风，严肃查处违法违纪案件，确保行政权力依法公正行使。

　　各位代表，回顾过去，发展成就令人鼓舞；展望未来，美好蓝图催人奋进。让我们更加紧密团结在以胡锦涛同志为总书记的党中央周围，深入贯彻落实科学发展观，在省委、省政府和市委的领导下，团结和依靠全市人民，解放思想、锐意进取，凝心聚力、奋力拼搏，为决战全面建设小康社会目标，在更高起点上推进龙岩科学发展、跨越发展，加快闽西老区崛起而努力奋斗，以优异成绩迎接党的十八大胜利召开！
